# Novel techniques to analyze dynamical properties of quantum chaos with peculiar evidence of hybrid systems confinement

**DOI:** 10.1038/s41598-024-61588-0

**Published:** 2024-05-14

**Authors:** Ghulam Bary, Waqar Ahmed, Riaz Ahmad, Shafiullah Niazai, Ilyas Khan

**Affiliations:** 1 Faculty of Science, Yibin University, Yibin, 644000 Sichuan China; 2 Key Laboratory of Computational Physics of Sichuan Province, Yibin University, Yibin, 644000 China; 3 Department of Bionanotechnology, Hanyang University, Ansan, 155-88 South Korea; 4 Department of Mathematics and Statistics, Nanjing University of Information Science and Technology, Nanjing, China; 5 Department of Mathematics, Education Faculty, Laghman University, Mehtarlam City, Laghman 2701 Afghanistan; 6 Department of Mathematics, College of Science Al-Zulfi, Majmaah University, Al Majmaah, 11952 Saudi Arabia; 7 Department of Mathematics, Saveetha School of Engineering, SIMATS, Chennai, Tamilnadu India

**Keywords:** Quantum chaos, Pattern formation, Hybrid systems, Chaotic parameters, Coherence, Mathematics and computing, Physics

## Abstract

Recent results demonstrate the dynamical peculiarities of the quantum chaos within the hybrid systems by chaotic parameters and probe the pattern formation under the influence of condensation. The complex dynamic behavior of the considered systems was determined with numerical simulation and presented an efficient technique that studied fractional systems comprising chaos-coherence fractions. The findings divulge the peculiar association between the coherence structure and the correlations at finite relative momenta. Thus the present study helps to explore the partially chaos hybrid systems in order to stimulate the experimental applications of nonlinear phenomena. The coherent-chaotic parameters can be measured by examining the chaos peculiarities that possess explicit relations with the condensations to demonstrate the environs of the physical systems. We investigate the influence of the multiplicities, chaos, momentum and temperature of the nonlinear system on the coherent-chaotic normalized correlations. The chaotic parameters are suppressed considerably with the coherence fraction and it appears numerically zero at maximum condensation and one at ideal chaos emissions. We procure that the meaningful parameters decrease significantly with the multiplicity of the nonlinear systems and increase with the momentum in the specified regimes. The identical multiplicity leads to contemplating the coherence and thus the normalized chaotic parameters within its spectacular influences exhibit significance worth contemplating in earnest. The findings underscore the significance of cogitating correlations in deciphering the nonlinear system characteristics and bestowing extraordinary perceptiveness into the convoluted essence of complex systems. The contemplated methodology can be applied to evaluating and analyzing the nonlinear systems and such an innovative approach computes the problems of celestial mechanics, heartbeats and chemical reactions in engineering and medical fields.

## Introduction

Interferometry introduces an effective tool to analyze the evolution of complex dynamical systems associated with the effects of long-term memory and examine the structure of the physical systems and the phase transitions at low temperature-momentum regimes which is an intriguing topic in nonlinear dynamics. The meaningful transitions reveal themselves via quantum chaos-coherence at specified temperatures with their influences depending on peculiar constituents such as spin, multiplicities, chaos and the degree of condensation fraction^1–3^. The normalized coherent-chaotic parameters are presented to demonstrate the nonlinear phenomena within the quantum interferences at zero relative momenta among multi-emitted identical particles produced in the nonlinear systems^4,5^. Such correlations disappear in the coherence emissions and occur in chaotic phenomena to explore the systems peculiarities. So the coherent-chaotic parameters results are associated with the chaos-coherent degree of the systems^6,7^. The specific relation between parameters and the ambiance for the considered nonlinear systems paves the path for appurtenant to compassionate the interferometry results^8–10^.

Additionally, the concept of quantum chaos-coherence eventuating from superpositions appears as an indispensable ingredient for quantum interferences and divergent correlations in order to explore physical systems. Numerous measures to evaluate the chaos at numerous temperature-momenta regimes have been investigated and such dynamic relations between quantum coherence and chaotic parameters possess intrinsic peculiarities about the nonlinear systems^11^. The emanated debris which pondered to the frequently emanated which is contemplated to employ the interferometry to demonstrate the pattern formation characteristics^12^. The substantial premise of the presented approach originated within the partially chaotic systems where the particles propagated with the evolution of stochastically emitted systems^13,14^.

Recently, the study of dynamics including stability, complexity, chaos and synchrony of dynamical systems with fractional derivatives has become an important research area. The results make it practicable to explore the parameters relative robustness and eradicate the complex factors to obtain specific elucidation about the system dimensions^15–18^. A versatile technique is presented to determine the characteristics and analyze the coherence dominance of the systems peculiarities under the quantum phenomenon^19,20^. The substantial principles have probed the momentum-temperature distributions of the systems with various variables which are defined at particular variables and the ramification of such condensation within the various particle manifestations specified^21^. Comparable normalized chaotic parameters determined the chaos degree of the nonlinear systems in order to investigate the wave propagation and identical particle field with differential equations^22–25^. The experimental results demonstrate the presence of coherence-chaos which suppresses the quantum interferences within the chaotic parameters significantly at finite relative momenta^26–31^.

Experimental elucidation shows that the considered systems possess debris of multiplicities in thousands and hundreds, respectively. Such strength produced condensation which gratified the suppression in chaotic phenomenon^32,33^. The rate at modest momenta and multiplicity have mutual relations with finite coherence-chaos fractions which diminishes the chaotic peculiarities within the exorbitant temperature. Hence the degree of coherence associated with the concomitant of systems asymmetries, chaotic signal and thus the distortions of the system can arise from dissipates of resonant, source geometries and fluxes^34–36^. Therefore the systems revealed are distinguished as chaotic if the coherence appears to be negligible which manifests the propensity to an equilibrium approach. Thus the significant inquisitiveness for partially coherent systems probed due to their implications and prevalence in numerous fields such as biology, engineering and physics. The impact of chaotic-coherent signals on the defined chaotic parameters in modern research would be intriguing for the analytical circumstances^37–39^.

We are explicative that the narrower systems exhibit higher condensation than wider systems at small relative momenta and temperature. Additionally, we find that the stochastic as well as velocity profiles within the small systems are more significant than the small nonlinear systems at various regimes. The present study also demonstrates that the velocity influences the chaotic parameters remarkably and thus the pattern formations are reduced significantly for higher-order correlations. Particularly, many researchers considered the primary correlations and the corresponding results influenced by various factors such as Fourier transform, temperature and flow peculiarities which urged us to explore the systems with nonlinear dynamics at higher order. Therefore we examined the various chaotic parameters at higher order which probed the intrinsic characteristics of the nonlinear systems. This study investigates the system peculiarities through density distributions, condensation and chaotic parameters. We have demonstrated momentum-dependent density distributions and normalized chaos parameters along with the structure analysis of the systems. The coherent and source-dependent captivated particle emissions parameters are interpreted to probe the pattern formation.

In this paper, the novel approaches within coherent-chaotic domains and multi-scale properties are examined for the nonlinear phenomena. In Section "Systems peculiarities with chaotic spectra", we demonstrate the system peculiarities with chaotic-coherent spectra. We explore the computing methods with various correlations, chaos parameters and their characteristics in Section "Computing methods and details". In Section "Solution of the problems", we elucidate the solution of the problems. Section "Results and discussion" described the findings and the partially chaotic emissions within the coherence influences. Finally, some concluding remarks are made in Section "Summary and conclusion".

## Systems peculiarities with chaotic spectra

In the present research work, nonlinear systems chaotic peculiarities have been frequently observed and contemplated in distinctive scientific fields particularly in meteorology, artificial intelligence and physics. Deterministic higher-order formulas investigate such nonlinear system distinctiveness which manifests as input parameter sensitivities, evanescent wave propagation and short dependability. Therefore the differential equations exhibit a powerful mathematical technique for exploring the system characteristics and dynamics with various parameters. We also demonstrate different levels of disintegration that can be generated with ground state density for the nonlinear systems while raising a possible impediment of powerful rotations. The considered approach computes the variations of the many-particle momentum densities, higher order nonlinear correlations and disintegration to explore the intrinsic peculiarities of the systems.

Numerical methods and mathematical theories for ground states demonstrate the applications of nonlinear dynamics of the considered phenomena and chaotic condensation characteristics. Extensions to GPE with an angular momentum rotation term for a rotating chaos-coherence with long-range anisotropic dipole-dipole interaction elucidates the nonlinear phenomena in dipolar coherence and coupled spin-orbit condensation-chaos peculiarities. Modern methodologies for the excited-ground states and their substantial characteristics play substantial contributions in nonlinear systems. Extensions of GPE within the rotation of angular momentum of the rotating coherence for the anisotropic dipole-dipole and coupled spin coherence exhibit significant applications. Therefore two components spin-orbital coupling condensation can be probed by the macroscopic wave function $\Psi _m:=\Psi _m(x,t)=(\Psi _{a}(x,t),\Psi _{b}(x,t))^T$ provided that the nonlinear system temperature $T_s$ smaller than the critical temperature and the meaningful evaluations governed by the following expressions

$$\begin{aligned} \begin{aligned}{}&i\hbar \partial _t \Psi _{a}=\left( -\frac{\hbar ^2}{2m}\nabla ^2 +V(x)+\frac{\hbar \tilde{\delta }}{2}+\frac{i\hbar \tilde{k}_0}{2m}\partial _x +\xi g_{11}|\Psi _{a}|^2+\xi g_{12}|\Psi _{b}|^2\right) \Psi _{a}+\frac{\hbar \tilde{\Omega }}{2} \Psi _{b}, \\&i\hbar \partial _t \Psi _{b}=\left( -\frac{\hbar ^2}{2m}\nabla ^2 +V(x)-\frac{\hbar \tilde{\delta }}{2}-\frac{i\hbar \tilde{k}_0}{2m}\partial _x +\xi g_{21}|\Psi _{a}|^2+\xi g_{22}|\Psi _{b}|^2\right) \Psi _{b}+\frac{\hbar \tilde{\Omega }}{2} \Psi _{a}, \end{aligned} \end{aligned}$$

where $\xi$ and $\tilde{k}_0$ represent the total multiplicity and the strength of the orbital coupling, respectively. $\tilde{\delta }$ and $\tilde{\Omega }$ describe the Raman transition and Rabi frequency to realize atomic Josephson junctions. Similarly, we can accomplish the dimensionless normalization condition with the reduction of 3D-2D-1D for the coupled condensation as

1 $$\begin{aligned} \begin{aligned}{}&i\partial _t \Psi _a=\left( -\frac{1}{2}\nabla ^2 +V(x)+\frac{\delta }{2}+i k_0\partial _x +\beta _{11}|\Psi _a|^2+\beta _{12}|\Psi _b|^2\right) \Psi _a+\frac{\Omega }{2} \Psi _b, \\&i\partial _t \Psi _b=\left( -\frac{1}{2}\nabla ^2 +V(x)-\frac{\delta }{2}-ik_0\partial _x +\beta _{21}|\Psi _a|^2+\beta _{22}|\Psi _b|^2\right) \Psi _b+\frac{\Omega }{2} \Psi _a. \end{aligned} \end{aligned}$$

The uniqueness as well as the existence of the ground state within the coupling appears accurate and thus the efficient numerical methods compute the coherence-chaos peculiarities. Therefore the problem with initial data corresponding to their numerical computation can be helpful by setting $\Psi _a(x,t)=\Phi _a(x,t) \textrm{exp}{i(\omega t+k_0x)}$ and $\Psi _b(x,t)=\Phi _b(x,t) \textrm{exp}{i(\omega t-k_0x)}$. The aforementioned equations under these conditions can be demonstrated as

2 $$\begin{aligned} \begin{aligned}{}&i\partial _t \Phi _a=\left( -\frac{1}{2}\nabla ^2 +V(x)+\delta +\beta _{11}|\Phi _a|^2+\beta _{12}|\Phi _b|^2\right) \Phi _a+\frac{\Omega }{2} e^{-i2k_0x}\Phi _b, \\&i\partial _t \Phi _b=\left( -\frac{1}{2}\nabla ^2 +V(x)+\beta _{21}|\Phi _a|^2+\beta _{22}|\Phi _b|^2\right) \Phi _b+\frac{\Omega }{2}e^{i2k_0x} \Phi _a. \end{aligned} \end{aligned}$$

Moreover, we present detailed multiple-systems elucidation of the processes in which associations originate during the rotationally confined coherence and thus the corresponding distribution function within the nonlinear dynamics can be demonstrated as

3 $$\begin{aligned} F_{be} = \left[ \textrm{exp}\left( \frac{E - E_g}{T_s}\right) - 1\right] ^{-1}. \end{aligned}$$

Here, the energy $E = \sqrt{m^{2}+p^{2}}$ represents the particles energy and such phenomena possesses an active role to study nonlinear dynamical characteristics with chaos-coherence across the complex systems where multiplicities exhibit substantial relation with temperature and momentum, respectively. Therefore the multiplicities can be expressed as

4 $$\begin{aligned} \xi \simeq \frac{\Omega _s g_n}{(2\pi )^3} \int d^{3}p\left[ \textrm{exp} \left( \frac{\sqrt{{p}^2 + m^2} - E_g}{T_s}\right) - 1\right] ^{-1}, \end{aligned}$$

where $g_n$ demonstrates the degeneracy and $\Omega _s$ expresses the volume of the considered system. The integral of the aforementioned equation becomes constant for the momentum *p* $\rightarrow$ 0 provided that $E_g \rightarrow m$. The meaningful results occur due to the statistical factor about the denominator singularity within the mathematical integration expressions $d^{3} {p}$. On the other hands, such term appear infinite when $E_g \rightarrow m$ for the zero momenta, i.e.,

5 $$\begin{aligned} \xi _{co} \simeq \frac{g_n}{ \textrm{exp} \left( \frac{m - E_g}{T_s}\right) - 1} \rightarrow \infty ~\textrm{for} ~E_g \rightarrow m. \end{aligned}$$

Particularly, thermodynamics demonstrate the specific peculiarities of the sources when the production of the system volume appeared as $\Omega _s \rightarrow \infty$ and thus the equation with the corresponding terms for *p* = 0 as well as *p* > 0 can be written as

6 $$\begin{aligned} \xi\simeq & {} \frac{g_n}{ \textrm{exp} \left( \frac{m - E_g}{T_s}\right) - 1} + \Omega _s \int \frac{d^3p}{(2\pi )^3} \frac{g_n}{\textrm{exp} \bigg (\frac{\sqrt{{p}^2 + m^2} - E_g}{T_s}\bigg ) - 1}\nonumber \\= & {} \sum _{n=1}^\infty \phi _z^n \frac{ e^{- n \varepsilon /T_s}(3-3e^{- n \varepsilon /T_s}+ e^{- 2n \varepsilon /T_s})}{(1-e^{- n \varepsilon /T_s})^3}, \end{aligned}$$

where $\phi _z$ and $\varepsilon$ represent the fugacity parameter and harmonic oscillator potential strength, respectively. Therefore the total multiplicity of the systems can be expressed in terms of the coherent as well as chaotic emissions

7 $$\begin{aligned} \Rightarrow \xi = \xi _{\textrm{co}} + \xi _{\textrm{ch}}, \end{aligned}$$

where the coherence fraction $\xi _{\textrm{co}}$ and chaotic fraction $\xi _{\textrm{ch}}$ demonstrate the multiplicities in coherence and the chaotic (excited) states, respectively. Thus the net computed multiplicity in terms of fugacity parameter can be elucidated as

8 $$\begin{aligned} \xi _t=\frac{\phi _z}{1-\phi _z}+\sum _{n=1}^\infty \phi _z^n \frac{ e^{- n \varepsilon /T_s}(3-3e^{- n\varepsilon /T_s}+ e^{- 2n \varepsilon /T_s})}{(1-e^{- n \varepsilon /T_s})^3}. \end{aligned}$$

In particular, the multiplicity $\xi$ and harmonic oscillator potential with temperature $\varepsilon /T_s$ are fixed and thus the configuration of the coherence condition can be computed numerically to examine the unknown $\phi _z$ by using Newton’s method. The value of $\xi _{co}$ and $\xi _{ch}$ explored with the determination of $\phi _z$ at various temperature regimes and thus the chaotic-coherent fractions of particles emitting sources can be determined by using aforementioned equations. Therefore the modified number density for coherent and chaotic emissions become

9 $$\begin{aligned} \rho _n\simeq & {} \frac{g_n}{\Omega _s \left( {\textrm{exp}} \left( \frac{m - E_g}{T_s}\right) - 1\right) } + \int \frac{d^3p}{(2\pi )^3} \frac{g_n}{\textrm{exp} \bigg (\frac{\sqrt{{p}^2 + m^2} - E_g}{T_s}\bigg ) - 1}\nonumber \\ \rho _n= & {} \rho _{\textrm{co}} + \rho _{\textrm{ch}}. \end{aligned}$$

We also observed that $\phi _z$ is touched to unity in the region of the strongly coherent at lower temperatures. Particularly, the parameter of fugacity $\phi _z$ at system temperatures $T_s=T=0$ assumes the mathematical expression

10 $$\begin{aligned} \phi _z(T_s=0)=\frac{\xi }{\xi +1}. \end{aligned}$$

For the given multiplicity $\xi$, the fugacity $\phi _z$ suppresses very slowly as the temperature of the system increases from minimum value $T_s$=0 and forms an obvious plateau provided that the considered temperature exists below the critical temperature. The larger the multiplicity $\xi$, the wider the plateau regime for the nonlinear systems as shown in the aforementioned figure. In the region of the transitional below the critical temperature within the substantial coherence fraction where significantly possible information obtains the considerable condensate fraction by measuring the value of $\phi _z$ which is close to the unity. Thus the chaotic emission can occur by setting the $\phi _z$ to unity and therefore we can illustrate the required mathematical expressions

11 $$\begin{aligned} \xi _t \sim \left( \frac{T_s}{\varepsilon } \right) ^3\sum _{n=1}^\infty e^{-n\varepsilon /2T_s}\left[ \frac{1}{n^3} +\frac{2(\varepsilon /T_s) }{n^2} +\frac{15(\varepsilon /T_s)^2}{8n} \right] . \end{aligned}$$

The used numerical method offers an efficient method for obtaining numerical solutions of chaotic systems with chaotic and coherent components. The influence of the coherence on the dynamics of the considered chaotic systems is explored mathematically and graphically. This study confirms that chaos does indeed exist in an ideal coherent system with fractional derivatives. Thus the pertinent condensate and chaotic fractions can be written in terms of $\xi _{co}(T_s)$ relatively with the parameters as the function of system temperature

12 $$\begin{aligned} \Psi _{co}(T_s)= & {} \frac{\xi _{co}}{\xi }=1-(T_s/T_h)^3 ~~~~~ \textrm{for}~~ T_s \le T_h, \end{aligned}$$

13 $$\begin{aligned} \Psi _{ch}(T_s)= & {} \frac{\xi _{ch}}{\xi }=(T_s/T_h)^3. \end{aligned}$$

**Figure 1:**
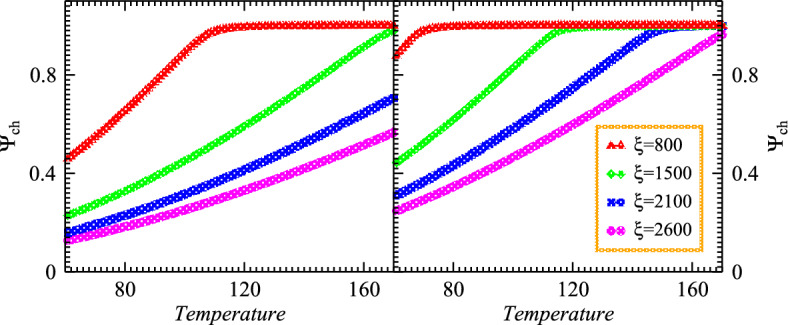
Chaotic emission spectra versus temperatures for the nonlinear systems with different source sizes and multiplicities.

In particular, we examine the dependence of various physical quantities on the considered behavior of the systems volume $\Omega _s$ at finite and infinite ranges. The transition of the phase in thermodynamics possesses the mathematical meaning at different values of $\Omega _s$. We need to start the systems with finite volume in order to define the required limit and the influences of the finite size are very important for determining the coherence-chaos characteristics. We use the different identities to acquire the expansion of density and thus one can write,

14 $$\begin{aligned}{}&\Delta \rho _s~=~\frac{3}{\pi ^2}\sqrt{\frac{m^3(m-\mu )^3}{2}}~ \int _0^{\infty }x^2dx\Big [\text {cth}\frac{\sqrt{m^2+2m(m-\mu )x^2}~-~m}{2T_s}~ \nonumber \\&\quad -\text {cth}\frac{\sqrt{m^2+2x^2m(m-\mu )}~-~\mu }{2T_s}\Big ]~\cong ~ \frac{3}{\pi ^2}\sqrt{\frac{m^3(m-\mu )^3}{2}}~ \int _0^{\infty }x^2dx\Big [\text {cth}\frac{x^2(m-\mu )}{2T_s}\nonumber \\&\quad - \text {cth}\frac{(x^2+1)(m-\mu )}{2T_s}\Big ] \cong \frac{6T_s}{\pi ^2}\sqrt{\frac{m^3(m-\mu )}{2}} \int _0^{\infty }-x^2dx\Big [\frac{1}{x^2+1}-\frac{1}{x^2}\Big ] =\frac{3T_s}{\pi }\sqrt{\frac{m^3(m-\mu )}{2}}~, \end{aligned}$$

where $\Delta \rho _s=\rho _s^*(T_s,m)~-~\rho _s^*(T_s,\mu )$. We need to discuss the more realistic picture with the system of finite volume and thus we consider the contribution from lower to higher quantum states for the total particle density as

15 $$\begin{aligned} \rho _s&\cong ~\frac{1}{\Omega _s}~\sum _{{ p},k}^{\infty } \langle \xi _{{ p},k} \rangle ~=~ 3\Big [\frac{1}{\Omega _s}~\frac{1}{\exp \left[ \left( m-\mu \right) /T_s\right] \;-\;1} \;+\;\frac{1}{\Omega _s}~\frac{6}{\exp \left[ \left( \sqrt{p_1^2+m^2}-\mu \right) /T_s\right] -1} \nonumber \\&\quad + \frac{1}{2\pi ^2}\int _{p_1}^{\infty }p^2dp~\frac{1}{\exp \left[ \left( \sqrt{p^2+m^2}-\mu \right) /T_s\right] -1}\Big ]~. \end{aligned}$$

The first and second terms of the aforementioned equation correspond to the lower momentum in the regime of p=0 and the first excited level, respectively at $p_1=2\pi \Omega _s^{-1/3}$ which possesses the degeneracy factor six. The third term in the r.h.s represents the approximate contribution from the higher levels when $p>p_1=2\pi \Omega _s^{-1/3}$. There is a particular relation $m=\mu$ for any finite volume $\Omega _s$ and forbidden to the infinite value of density at zero momentum level. Therefore we expect non zero density at $T_s<T_{cr}$ at the zero momentum and this needs $(m-\mu )/T_s\equiv \delta \propto \Omega _s^{-1}$ at $\Omega _s\rightarrow \infty$. Hence the density at a lower momentum level can be determined as

16 $$\begin{aligned} \rho _l=\frac{18~\Omega _s^{-1}}{\exp \left[ \left( \sqrt{p_1^2+m^2}-\mu \right) /T_s\right] -1} \cong \frac{18~\Omega _s^{-1}}{p^2_1/(2mT_s)~+~\delta }~\propto ~\frac{\Omega _s^{-1}}{\Omega _s^{-2/3}}~=~\Omega _s^{-1/3}~. \end{aligned}$$

Such intriguing and meaningful expression becomes zero at $\Omega \rightarrow \infty$ and the second term in the second last equation can be neglected in order to use it in pragmatic applications. Moreover, the variance of the particle multiplicity fluctuations at finite $\Omega _s$ can be demonstrated as:

17 $$\begin{aligned}{}&\langle \Delta \xi ^2\rangle ~\equiv ~ \langle \left( \xi ~-~\langle \xi \rangle \right) ^2\rangle ~=~\sum _{{ p},k}\langle \varrho _{{ p},k}\rangle (1+\langle \varrho _{{ p},k}\rangle ) \;\cong \; 3\left[ \frac{1}{\exp \left[ \left( m-\mu \right) /T_s\right] \;-\;1} \right. \nonumber \\&\left. \quad +\frac{1}{\left\{ \exp \left[ \left( m-\mu \right) /T_s\right] \;-\;1\right\} ^2} \;+\; \frac{\Omega _s}{2\pi ^2}\int _{0}^{\infty }p^2dp\; \frac{\exp \left[ \left( \sqrt{p^2+m^2}-\mu \right) /T_s\right] }{\left\{ \exp \left[ \left( \sqrt{p^2+m^2}-\mu \right) /T_s\right] \;-\;1\right\} ^2}\right] ~~. \end{aligned}$$

The given expression elucidates that the first two terms correspond to the lower level zero momenta and the last term appears when momentum is greater than zero. Due to the massive applications and relations of nonlinear dynamics in many different areas such as aeronautical, mechanical structure and control systems have been applied almost one century ago and have grown explosively up to now. The research in the experimental areas plays an active role and extensive demand due to technological progress in nonlinear dynamics.

**Figure 2:**
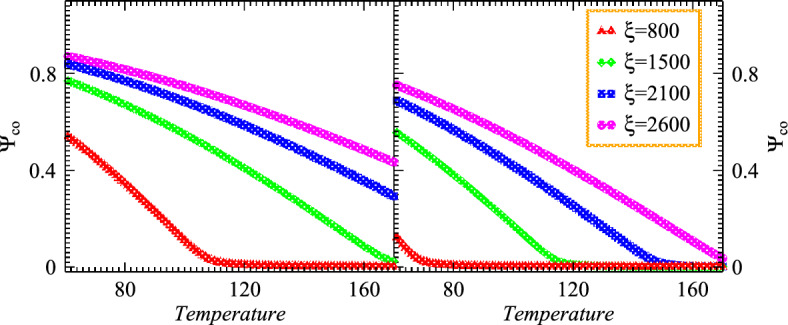
Coherent emission spectrum of the nonlinear systems versus temperature which possess different sizes and multiplicities.

Fig. 1 left and right panels illustrate the chaotic spectrum versus temperature of the examined nonlinear systems which are dilatant and cool gradually after their production. The particulars of the considered systems probed within the measured parameters 0.35 and 0.40 as demonstrated with two horizontal left and right panels for small and large sources, respectively. The multiplicities are fixed within the limit of $\xi =700-2500$ and the evolution of coherence as well as chaotic emissions explored at different temperature regimes. One can observe that the chaotic emissions represent the dominance gradually with escalating temperature profile at constant $\xi$ and system sizes. Such intriguing and meaningful spectra touch the chaotic limit at higher temperature as shown by an upper top dotted red dotted line. The lower multiplicities at moderate temperatures probed the nonlinear systems with partial chaotic emissions. On the other hand, the chaotic emission decreased continuously with increasing the density of emitted particles within the system at particular lower temperature regimes. Therefore the reducing density extensively enhanced the chaotic spectrum with the presence of thermal systems. Therefore the chaotic emissions exhibit pertinent relations with the energy and temperature of the nonlinear systems.

In addition, Fig. 2 shows the coherence emission diagrams of the nonlinear systems regarding the parameter $\xi$ with the small and large sizes, respectively for the considered systems that cool and expand gradually. The coherent emissions spectra demonstrate considerable suppression by increasing the temperature at constant size and multiplicities $\xi$. One can note that the condensation increases with the escalation of multiplicities and vice versa under the influence of condensation. Such an intriguing fraction appeared slightly below the coherent limit as shown with both panels at lower temperatures. On the other hand, the coherence increases continuously with increasing the density for the small system at particular lower temperature regimes than that of the large system as shown in the right panel. Moreover, it is meaningful to mention that the reduced particle density remarkably suppresses the coherent emissions with the absence of chaotic particles. Such interesting results explore the nonlinear systems that exhibit coherent components and possess specific relations with the temperature of the pertinent sources.

**Figure 3:**
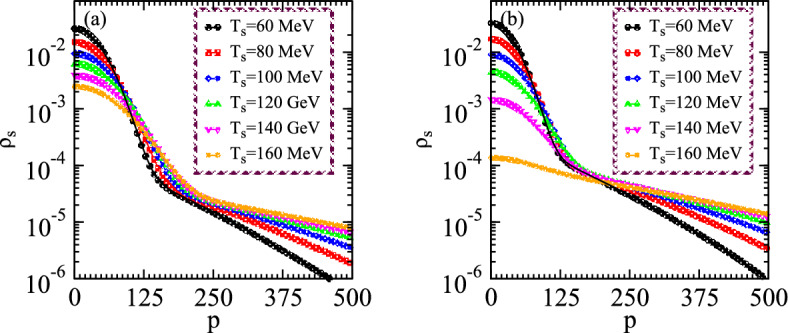
Density distributions versus momentum within different source sizes at constant multiplicities of the considered nonlinear systems with the variation of temperature.

The current research explored the acquired approaches for extracting the coherence component from the emission constrained by the emergence of partial nonlinear phenomena. In the presence of coherence fraction, the response of the complex systems probed with quantum statistics and eventual interactions significance influences the quantum correlated functions at finite temperature. For massively expanded systems, the acquisition of the coherence image from various correlation functions and particle emissions is explored in detail.

In particular, phase transition exhibits condensation due to the coherence effect with the existence of multiplicities in the same quantum level. Therefore mathematical investigations of the diluted matter in traps are presented to explore the systems intrinsic peculiarities. The basic characteristics of the chaos-coherence of multiplicities are explained using the theories of mean-field under nonlinear dynamics. The volumetric profiles, the energy content of the ground state variations, the combined fluctuations, the dynamics of the rate of expansion, the condensate percentage and the dynamics of the thermodynamic characteristics are the aspects of such complex systems. The examined systems shell dimension and temperature scales show scaling tendencies at the thermodynamics limits. Although the matter is diluted with the noninteraction which possesses a significant impact on both the static and dynamic characteristics of the nonlinear systems and thus the mean-field potential estimates the existing data from experiments very well. From these computations, one can realize that the considered system at higher temperatures seems chaotic at finite whole multiplicities.

Figs. 3(a) and 3(b) demonstrate the distribution of densities versus momenta for the small and large systems at different temperatures. One can note that the density distribution decreases with increasing the momenta at constant temperature and thus these distributions are suppressed remarkably with increasing the temperature at constant momentum. Such intriguing results explore that the particles with high momenta emanated from the excited states which possess chaotic emissions characteristics. The considered nonlinear system expands and cools with the interval of time to explore the characteristics of the chaotic-coherence peculiarities. The right panel shows the system which possesses constant multiplicities and the distribution for the small system has an obvious enhancement in the small momenta regime under the existence of coherence than that of the large one which also possesses identical particle density. The reason for such rising densities at small momenta is quite obvious because the small source has a fine degree of source coherence emission due to condensation even at the higher temperature. However, the large system appeared almost completely chaotic at the specified temperature than that of the small system. Particularly, the density distributions show the two tiered structures caused by the condensation of the systems lower temperatures and thus the corresponding distributions are pretty wider as compared to the large system which explores the partially coherent characteristics within the influence of coherence emission.

**Figure 4:**
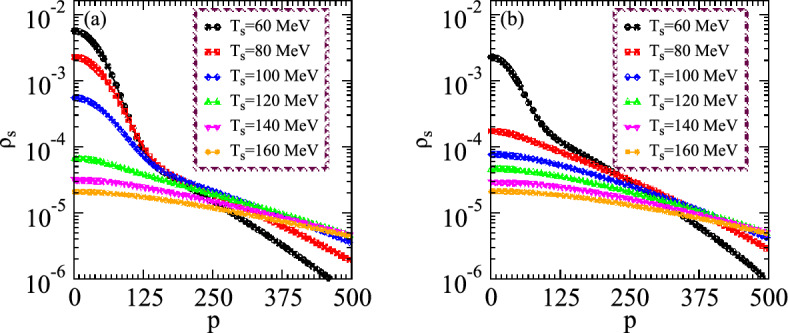
Density distributions versus momentum for two certain values of the source size parameters at constant particle multiplicities of the nonlinear systems with the variation of temperature.

In Fig. 4, we initially examine the density distributions of the two nonlinear systems with lower multiplicity in the partially chaos medium with the variation of momenta for the small and large systems as shown in the left and right panels, respectively. According to the aforementioned distribution equations, the distributions of typical particulate grow as its temperature decreases in the whole regimes of momenta. The multiplicity influences the density distribution at the small temperature regimes in the condensed medium and shows the enhancement by lowering the temperatures in the presence of coherence. Additionally, during elevated chemical potential values, we observed that the multiplicity increases for the condensation with the incorporation of a chemical perspective and thus the system densities increase significantly at the specified regimes. The plots probed that the investigation of chaos with definite chemical susceptibility exhibits a substantial role in the nonlinear phenomena. Hence the density of the systems increases with decreasing the source size at the fixed temperature as shown in the right and left panels, respectively.

Such consequences arise since the small nonlinear system possesses more condensation than the large one because the small system contains massive energy gaps which makes the potential barrier for existing particles to cross it. Therefore these multiplicities at the the ground state form condensation which influences the chaotic peculiarities. Specifically, the right panel demonstrates that the density distribution suppresses significantly for the large system than the small size in the whole regimes of temperature due to the presence of chaotic emission for the large expanding system. The intriguing results probed the complex system structure at various temperature regimes and also explored the chaos-coherence characteristics at distinct multiplicities as well as the system size to examine the collective dynamical behavior in distinguishable configurations and symmetries. It means that the chaos emissions are persistent with the large systems at a higher temperature which comprises hundreds of particle multiplicities in the expanding system and such coherence-chaos nonlinear systems can be used in engineering and medical applications.

## Computing methods and details

In this section, we explored the dynamical behavior with the collective dynamics of the nonlinear systems in the presence of partial chaotic peculiarities and computed the correlations along their corresponding coherent-chaotic parameters. Such parameters possess explicit relations with the condensations to demonstrate the systems and investigate the influence of the multiplicities, momentum, chaos and temperature of the system on the coherent-chaotic normalized correlations. Thus the nonlinear dynamical analysis represents the quantum mechanical peculiarities to probe the intrinsic characteristics of the systems and therefore the complex complications can be elucidated through nonlinear dynamics. We demonstrate the particularism of low-mass detected particles that possess sufficient energy to proliferate from production to detection. It is imperative to investigate the nonlinear systems using noble phenomena and acquire pragmatic techniques instead of the conventional elucidation of coherence-chaotic systems. Therefore more inquisitiveness has been considered to partially coherent systems due to their implications and prevalence in numerous fields such as biology, engineering, artificial intelligence, and medical physics.

**Figure 5:**
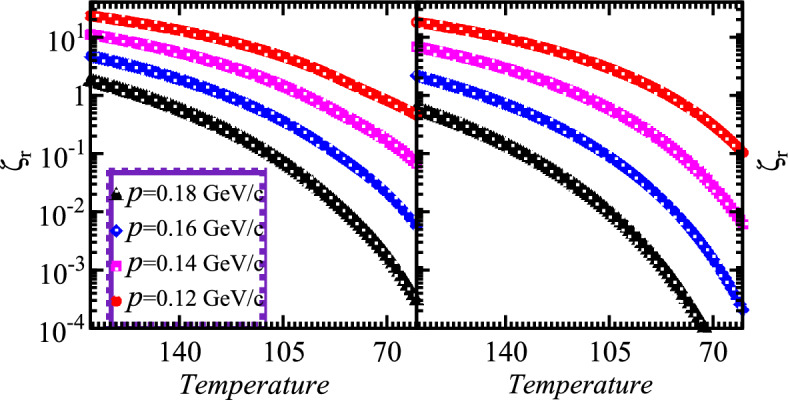
$\zeta _r$ versus temperatures for nonlinear systems possess low multiplicities and partially chaotic emissions at different momenta regimes.

Researchers and scientists have been initiated to deflect their focus from nonlinear systems exploration in recent years to recognizing the importance of understanding nonlinear phenomena within coherence and chaotic wave propagation in specific physical systems. This elaborating interest in the considered systems underscores the peregrination of nonlinear dynamics which exhibits the scientific applications. The current research goal focuses on investigating the distinctive characteristics of the systems that are generated as a result of the consequences of wave propagation. Quantum computationally normalized chaotic variables within the systems enable structural stability and dynamical mathematical equations describe the intrinsic characteristics of the extended system along with the multiplicities at finite temperature. We describe the nonlinear system modeling culminates for partially thermodynamic structures with momentum dependence and propose that the emanated systems contained coherence-chaos collections that perform as partially coherent sources at finite momentum and temperature. Thus the well-known relation between the ratio of condensing to whole density becomes as $\zeta _r=\xi |u_{c}|^2/f(p)$.

Specifically, $\zeta _r$ demonstrates the specific relation about the relative probability with the total momentum density at different momenta and temperature regimes. The system exhibits the coherence constituent of the partially condensation possesses the relative distribution $\zeta _r$ at wide momenta which suppresses significantly with increasing momentum because the coherence density decreases considerably than the density distribution f(p) with an increase in the momenta at finite temperature regimes.

Fig. 5 elucidates $\zeta _r$ versus temperatures at various momenta for the nonlinear systems that possess minimum multiplicities at different system sizes. Thus the left and right panels describe the variation of $\zeta _r$ with momenta of particles for small and large systems, respectively. One can observe an obvious enhancement at higher temperature regimes with the variation of the momenta and show the suppression at lower temperature regimes. The intriguing results indicate that the $\zeta _r$ increases gradually with increasing the temperature at constant source size and mean momenta. Such parameters described that the values of $\zeta _r$ also probe an enhancement with increasing the size of the nonlinear systems at constant temperature and momenta domains because the total density distribution becomes wider within the narrow radius whenever the explored systems are accompanied at the higher temperature. Results possess the absence of sharp changes in $\zeta _r$ as the momentum increases for the entirely uncondensed emissions which can be seen at lower momentum when p= 0.12 GeV. The right panel shows more enhancement than the left panel since the large system exhibits wider size and narrower energy gaps which provide a convenient environment to overcome these massive energy gaps. Therefore distributions of p appeared wide for small nonlinear systems under specific temperature which influences $\zeta _r$.

Nonlinear dynamical analysis at higher order possesses significantly more information than the analysis of lower order and such computations with chaos-coherence peculiarities dominated the chaotic coefficients considerably. Therefore numerous higher-order dynamical analyses carry more inherent aspects about systems than lower ones which prompted to probe of the nonlinear phenomena and the peculiarities of the system which changed with the intrinsic geometry of the nonlinear systems. The momentum-dependent analysis via relative momenta is investigated because such analysis contains the advancement and prevalence of the coherent-chaotic transition temperature. In this research, the density matrices appear as the substantial constituents in phase space for the partial chaotic phenomena. Hence the analysis of the second, third, and fourth order are explored by contemplating the mutual relation of wave propagation within the considered systems. The numerical method contained an efficient technique for obtaining numerical solutions of fractional chaotic systems with variable-order exponents:

18 $$\begin{aligned} \Theta _4(q_4)= & {} 1 + \Upsilon _3(p_\nu ,p_\mu , p_\sigma )+\Upsilon _2(p_\nu ,p_\mu )+\Upsilon _{2p}(p_\nu ,p_\mu ) +\Upsilon _4(p_\nu ,p_\mu ,p_\sigma ,p_\psi ), \end{aligned}$$

19 $$\begin{aligned} \theta _4(q_4)= & {} 1+\Upsilon _4(p_\nu ,p_\mu ,p_\sigma ,p_\psi ). \end{aligned}$$

The influences of such ingredients present in the chaotic systems due to the variation of phase and such nonlinear phenomena depend on the relative momenta in the specific regimes to explore the nonlinear systems. The effect of interferences disappears for the system with coherent peculiarities under the isolation of phase change and therefore the chaotic parameters show their independence about the relative momenta.

In particular, higher-density matrices can be examined via single-particle density matrices to explore the characteristics of nonlinear systems. Hence the normalized chaotic parameter computed by combining the aforementioned density mixtures and the evaluation can be demonstrated with different equations to understand the nonlinear phenomena. We need an inversion formula which involves the chaos-coherence peculiarities as given below

20 $$\begin{aligned}{} & {} f^{(1)}(p_2,p_1).f^{(1)}(p_3,p_2).f^{(1)}(p_4,p_3). f^{(1)}(p_4,p_1)=\mathbbm {k}_\alpha (1234)+\mathbbm {k}_\beta (1234)+\mathbbm {k}_\gamma (1234)\nonumber \\{} & {} \quad +3n_{c}^4 |u_{c}(p_1) u_{c}(p_2) u_{c}(p_3)u_{c}(p_4)|^2. \end{aligned}$$

where symbols have their usual meanings $\mathbbm {k}_\alpha (1234)$, $\mathbbm {k}_\beta (1234)$ and $\mathbbm {k}_\gamma (1234)$ represent the emissions from chaotic-coherence components, respectively. Such expression can elucidate the nonlinear phenomena at finite momenta and can be expressions as

21 $$\begin{aligned}{} & {} \mathbbm {k}_\alpha (1234)=f_{ch}^{(1)}(p_2,p_1).f_{c}^{(1)}(p_3,p_2).f_{ch}^{(1)}(p_4,p_3). f_{ch}^{(1)}(p_4,p_1)\nonumber \\{} & {} \quad +f_{c}^{(1)}(p_2,p_1).f_{ch}^{(1)}(p_3,p_2).f_{ch}^{(1)}(p_4,p_3). f_{ch}^{(1)}(p_4,p_1)\nonumber \\{} & {} \quad +f_{ch}^{(1)}(p_2,p_1).f_{ch}^{(1)}(p_3,p_2).f_{c}^{(1)}(p_4,p_3). f_{ch}^{(1)}(p_4,p_1)\nonumber \\{} & {} \quad +f_{ch}^{(1)}(p_2,p_1).f_{ch}^{(1)}(p_3,p_2).f_{ch}^{(1)}(p_4,p_3). f_{c}^{(1)}(p_4,p_1), \end{aligned}$$

22 $$\begin{aligned}{} & {} \mathbbm {k}_\beta (1234)=-2f^{(1)}(p_4,p_1) n_{c}^3 u_{c}(p_1)u_{c}(p_4)|u_{c}(p_2)u_{c}(p_3)|^2\nonumber \\{} & {} \quad -2f^{(1)}(p_2,p_1) n_{c}^3 u_{c}(p_1)u_{c}(p_2)|u_{c}(p_3)u_{c}(p_4)|^2\nonumber \\{} & {} \quad -2f^{(1)}(p_4,p_3) n_{c}^3 u_{c}(p_3)u_{c}(p_4)|u_{c}(p_1)u_{c}(p_2)|^2\nonumber \\{} & {} \quad -2f^{(1)}(p_3,p_2) n_{c}^3u_(p_2) u_{c}(p_3)|u_{c}(p_1)u_{c}(p_4)|^2\nonumber \\{} & {} \quad -2f^{(1)}(p_4,p_3) n_{c}^3u_{c}(p_3)u_{c}(p_4)|u_{c}(p_1)u_{c}(p_2)|^2\nonumber \\{} & {} \quad \times \left[ f_{ch}^{(1)}(p_2,p_1).f_{ch}^{(1)}(p_3,p_2).f_{ch}^{(1)}(p_4,p_3). f_{ch}^{(1)}(p_4,p_1)\right] , \end{aligned}$$

23 $$\begin{aligned}{} & {} \mathbbm {k}_\gamma (1234)=f^{(1)}(p_4,p_1).f^{(1)}(p_4,p_3) n_{c}^2 u_{c}(p_1) u_{c}(p_3)|u_{c}(p_2)|^2\nonumber \\{} & {} \quad +f^{(1)}(p_4,p_1).f^{(1)}(p_2,p_1) n_{c}^2 u_{c}(p_2) u_{c}(p_4)|u_{c}(p_3)|^2 \nonumber \\{} & {} \quad +f^{(1)}(p_4,p_1).f^{(1)}(p_3,p_2) n_{c}^2 u_{c}(p_1) u_{c}(p_2)u_{c}(p_3)u_{c}(p_4)\nonumber \\{} & {} \quad +f^{(1)}(p_2,p_1).f^{(1)}(p_4,p_3) n_{c}^2 u_{c}(p_1) u_{c}(p_2)u_{c}(p_3)u_{c}(p_4)\nonumber \\{} & {} \quad +f^{(1)}(p_3,p_2).f^{(1)}(p_4,p_3) n_{c}^2 u_{c}(p_2) u_{c}(p_4)|u_{c}(p_1)|^2\nonumber \\{} & {} \quad +f^{(1)}(p_2,p_1).f^{(1)}(p_3,p_2) n_{c}^2 u_{c}(p_1)u_{c}(p_3)|u_{c}(p_4)|^2. \end{aligned}$$

In addition, the required interferences with density matrices demonstrate the nonlinear phenomenon in a particular paradigm and thus the collective phenomena within the nonlinear dynamics are substantially influenced by the coherence and their corresponding chaotic parameters are highly dependent on the coherent fraction of the nonlinear systems. The coherence exhibits remarkable influences on higher level correlations than those of the primary interferences and the coherence at higher multiplicity reflect significant impacts at particular temperature-momenta regimes. The combined effect of the nonlinear systems can be manipulated for the cumulant summation of the aforementioned expressions and the meaningful higher order chaotic correlator $\Upsilon _{4}(p_4,p_3,p_2,p_1)$ can be demonstrated in terms of the density matrices,

24 $$\begin{aligned}{} & {} \Upsilon _{4}(p_4,p_3,p_2,p_1)=f^{(1)}(p_2,p_1). f^{(1)}(p_3,p_2). f^{(1)}(p_4,p_3). f^{(1)}(p_4,p_1)\nonumber \\{} & {} \quad +f^{(1)}(p_2,p_1).f^{(1)}(p_4,p_2).f^{(1)}(p_4,p_3). f^{(1)}(p_3,p_1)\nonumber \\{} & {} \quad +f^{(1)}(p_3,p_1).f^{(1)}(p_3,p_2).f^{(1)}(p_4,p_1). f^{(1)}(p_4,p_2). \end{aligned}$$

These results can be expressed in the more sophisticated form for the normalized chaotic correlations at various relative momenta and temperature. The summarized equations play an accomplished contribution through possible demonstrations to examine the chaotic as well as the coherence of the nonlinear systems. Moreover, We examine the implications of nonlinear systems within the wave propagation, pattern formation and such a singular formula reveals the contribution of two, three, and fourth order with experimental applications. The fractions of chaos and coherence can be computed using the available data of the normalized chaotic correlations at the fourth order. The presented mathematical scenario appeared under the circumstances when the nonlinear systems entirely chaotic, partially or ideally coherent at numerous momentum regimes to demonstrate the applications of nonlinear science in complex networks and artificial intelligence. Particularly, we discussed the meaningful performance of chaotic-coherent investigations and found that they perform significantly better than classical probes at finite temperatures. Novel capacities for inertial chaos, acoustic pattern formation and investigations for the new nonlinear phenomena might be made possible by the higher-order nonlinear dynamical analysis.

In particular, the considered technique examined the chaos-coherence properties of the nonlinear systems through interferences and thus the higher-order chaotic parameters explore the nonlinear systems chaos-coherence peculiarities. The lower order chaotic attractor with the single particles obtained and such multiplication represented to demonstrate the collective phenomena for physical systems to develop the entanglements within the nonlinear systems. Thus the density factor appeared significant in probing the corresponding results and the chaotic-coherent components of the distribution functions associated with the evaluated multiplicities and temperatures. The influence of the variable-order exponents on the dynamics of the considered chaotic systems also explored. This current section confirms that chaos does indeed exist in fractional systems with variable-order fractional derivatives.

## Solution of the problems

This section is concerned with the dynamic behavior of the nonlinear systems that span an oscillating loop with temperatures and momentum oscillations in the presence of quantum consequences. The mitigation technique of density correlations formed for distinct collective flowing phenomena characteristics which explore the temperature description, and the possibilities for density formulae. Therefore the hydrodynamics probed the transformation between the cold-hot paradigm that started by the transition during the expansion of emitting systems depicted by computational techniques that display statistical information of chaotic droplets. Our method anticipates an important scientific relationship between the pattern formation of phase structure within the general effectiveness and the crossover level. The emerging phase can have a partially chaotic distribution with temperatures determined by the speed of the phase transitions in the nonlinear phenomena. The presented phase also shows the intriguing applications of collective phenomena in chemical and physical systems. We develop the normalization intercepting for the combination of systems utilizing specific numerical techniques to evaluate the modification of the system which allows us to examine the substantial peculiarities of the systems.

The normalization stochastic characteristic can be demonstrated through mathematical computations and thus the aforementioned equation determines the intrinsic characteristics of the chaotic percentage under the partially chaotic factors at finite momenta. The associated normalized chaotic parameter reveals chaotic limit six within the presence of absolute chaotic characteristics. The presented approach exhibits peculiarities about the chaotic and coherent producers across the nonlinear systems investigations. Hence the normalized chaotic parameter can be elucidated as

25 $$\begin{aligned} w_4{(q_4)}=\frac{\Upsilon _{4}(p_4,p_3,p_2,p_1)}{\sqrt{\Omega _2(p_4,p_1)\Omega _2(p_4,p_3)\Omega _2(p_3,p_2)\Omega _2(p_2,p_1)}}, \end{aligned}$$

where $\Upsilon _{4}(p_4,p_3,p_2,p_1)$ and $\Omega _2(p_4,p_1)\Omega _2(p_4,p_3)\Omega _2(p_3,p_2)\Omega _2(p_2,p_1)$ represent the formulation for genuine four particles and the primary correlators interferences, respectively. Thus the higher order normalized chaotic parameter becomes

26 $$\begin{aligned} w_4{(p)}=2.\frac{\left[ 3-18\left[ \upsilon _{c}(p)\right] ^2 +24\left[ \upsilon _{c}(p)\right] ^3-9\left[ \upsilon _{c}(p)\right] ^4\right] }{\sqrt{\left[ 1-\left[ \upsilon _{c}(p)\right] ^2\right] ^4}}. \end{aligned}$$

In particular, we probe the coherence-chaotic characteristics to determine the intrinsic aspects of the nonlinear systems and thus the normalized correlators at the intercept contained meaningful methodologies at fourth-order paradigms. Therefore the coherent fractions can be explicated as $f_{c}(p)/f(p)=\upsilon _{c}(p)=1-\upsilon _{c}(p)$ and such expression of the coherence-chaotic constituents demonstrate the system specific dimensions at finite temperature. The exact formulation of the chaos at the fourth-order parameter can be derived by manipulating the values for both the chaos and coherence percentage in the computations under the collective phenomena. The coherent-chaotic fragments examined significantly using partial differential equations for the normalizing correlation chaotic parameter and certain expressions describe the meaningful and intriguing fraction at the intercept. The aforementioned mathematical expressions can be expressed in substantial form as

27 $$\begin{aligned} \Pi _4^{p}=\frac{\upsilon _{(ch)}(p)\left[ 4-3\upsilon _{(ch)}(p)\right] }{\left[ 2-\upsilon _{(ch)}(p)\right] ^2}. \end{aligned}$$

One can note that the interesting results illustrate the partially chaotic-coherent characteristics at different momenta and the corresponding chaotic gauge parameter becomes unity $\upsilon _{(ch)}(p)=1$. Therefore the analogous results suggest the normalized chaotic parameter $\Pi _4^{p}=1$ and determine the perfect coherent sources that exhibit $\Pi _4^{p}=0$. It means that the current research can explore the chaotic-coherent distinctions with remarkable equations and possess applications in chemical, biological and physical systems.

Moreover, it is more intriguing to introduce the standardized correlation coefficients for the granular nonlinear phenomena corresponding to the chaotic parameter which can be probed in the particulate ejected nonlinear systems as follows: $\beta _{2}^{g}=(1-\upsilon _r)$, $\beta _{3}^{g}={(1-2\upsilon _r)}/{\sqrt{(1-\upsilon _r)}}$, $\beta _{4}^{g}={(1-2\upsilon _r)(1-3\upsilon _r)}/{(1-\upsilon _r)}$. The significant and interesting demonstration mentions that the inverse of the multiplicity droplets represented by $\upsilon _r$ plays a key role during the search for the considered system coherent emissions. Thus the substantial coherence particle effluent systems normalizing chaotic parameter intercept for two, three, and four particles are expressed within coherent peculiarities as $\beta _2^{p}={1+2\upsilon _c}/{(1+\upsilon _c)^{2}}$, $\beta _3^{p}={(1+3\upsilon _c)}/{\sqrt{(1+2\upsilon _c)^{3}}}$, $\beta _4^{p}={1+4\upsilon _c}/{(1+2\upsilon _c)^{2}}$. It should be obvious that the parameter of coherence $\mu _c$ quantifies the ratio of coherent to chaotic fractions to explore the aspects of the hybrid systems.

**Table 1:** Consequences of the coherence-chaotic fraction on four, three and two particles normalized chaotic parameters with the hybrid nonlinear systems as partial coherence sources.

$\upsilon _c$	$\Psi _{co}$	chaoticity parameter $\beta _2^{p}$	normalize parameter $\beta _3^{p}$normalize chaotic parameter $\beta _4^{p}$	$\Psi _{ch}$	
0.10	0.09	0.96	0.988	0.97	0.91
0.15	0.13	0.96	0.977	0.95	0.87
0.24	0.19	0.96	0.96	0.89	0.81
0.46	0.31	0.90	0.89	0.77	0.69
0.54	0.35	0.88	0.88	0.73	0.65
0.81	0.44	0.80	0.81	0.61	0.56
1	0.50	0.75	0.77	0.56	0.50

Table 1 represents the precise ambiance of the chaos-coherent debris of the nonlinear systems at particular energies. It is evident that the explored characteristics of the partially coherent spectrum and the corresponding parameters $\beta _2^{p}$, $\beta _3^{p}$ and $\beta _4^{p}$ vary gradually in different regimes with the deviation of $\upsilon _c$. Thus the physical systems are composed of the partially chaotic and coherent sub systems and the peculiarities of such sources disseminated the conglomerated and intangible special degree that probed the system evolution phenomenon. The expressions show that the component of coherent coefficient $\upsilon _c$ suppresses the intercept of the four particles evidently which demonstrates the influence of coherence in the presence of chaotic-coherent radiations. Therefore it substantiates gravely in the intercept form within the normalized chaotic parameters which examine the chaotic limit to explore the nonlinear systems.

In particular, the influences of $\upsilon _c$ variation on the examined multitude intercepts determined the particulars of debris rather than the simple chaotic normalization factors. The results appeared at higher order intercepts $\beta _4^{p}$ which exhibit the signification discretion than those of $\beta _2^{p}$ for specified $\upsilon _c$. The data of normalized chaotic parameters intercept $\beta _4^{p}$ enthrall lower statistics than $\beta _2^{p}$ which is possibly congruous with the experimental calculated results. It means that the normalized chaotic parameter at higher order comprised peculiar characteristics than elementary order to explicate the coherent-chaotic data for the nonlinear phenomena.

**Table 2:** Two, three, and four-particle coherence-chaos parameters associated with droplets through the granular partially chaotic nonlinear systems as isotropically coherence particles aminated droplets.

$\upsilon _r$	$\Lambda _c$	chaoticity parameter $\beta _2^{g}$	normalize parameter $\beta _3^{g}$	normalize parameter $\beta _4^{g}$
0.005	200	0.995	0.992	0.980
0.010	100	0.990	0.955	0.960
0.012	80	0.980	0.970	0.950
0.040	30	0.960	0.950	0.870
0.050	20	0.950	0.920	0.800
0.070	15	0.930	0.900	0.740
0.100	10	0.900	0.840	0.620
0.130	8	0.870	0.800	0.530
0.200	5	0.800	0.670	0.300
0.250	4	0.750	0.580	0.170

Table 2 describes the nonlinear systems that consist of small dispersed droplets with multiplicity $\Lambda _c$ which has attracted considerable attention due to its unique properties and wide variety of applications in physical and biological sciences. The results of two, three and four chaotic intercepts increased with increasing $\Lambda _c$ leading to a larger possibility of emissions from separate drops. When such systems cool down to a particular temperature with the subsequent formation of granular droplets then they make a phase transition provided that the transition of phase appears as first-order. We investigate these influences on the normalized correlation functions and their corresponding intercepts of chaotic parameters in order to explore the system peculiarities. The results elucidate that the normalized chaotic parameters intercept rises significantly with the multiplication of the droplets because the manifestation of copious numbers of $\Lambda _c$ exhibits a higher probability of emissions from the distinct droplets. It is therefore obvious that the normalization of chaotic variables yielded a lower value within the presence of coherence than the two-particle intercepting parameter which is compatible with the research findings. We can conclude that the granularity structure paradigm appears comprehensive and explores the scientific results for chaotic parameters which measures the degree of coherent-chaotic constituents remarkably.

## Results and discussion

New kinds of chaotic parameters for fractional-order chaotic systems are introduced in the following sections. We extended some familiar chaotic systems by implementing the generalized higher-order methodologies. We probed the particularism that possesses sufficient energy to proliferate from production to the observation points and imperative to explore the nonlinear systems using the interferometric techniques in nonlinear phenomenon. The considered systems are distinguished as chaotic if the coherence appears to be negligible which reveals the propensity at various relative momentum. Therefore more inquisitiveness investigated partially chaotic systems due to their implications and prevalence in numerous fields such as biology, engineering and physics. Researchers and scientists have been initiated to deflect their focus from space-time exploration in recent years to recognizing the importance of nonlinear phenomena with coherence and chaotic wave propagation in specific systems.

The significance of coherence on catastrophic generations is elucidated through correlation diagrams, tables and thus the normalizing degree for nonlinear systems demonstrate the implications of constituent within partially chaotic fluxes. When the coherent influence prevails the systems then the corresponding computations contains the flow of matter that expand over an oscillating track with density and temperature perturbations. The resultant phase fluctuations of the swift Fourier transform can be controlled by the ratio of the combined effects of lower order to the progressively ambivalence interferences of fourth order.

**Figure 6:**
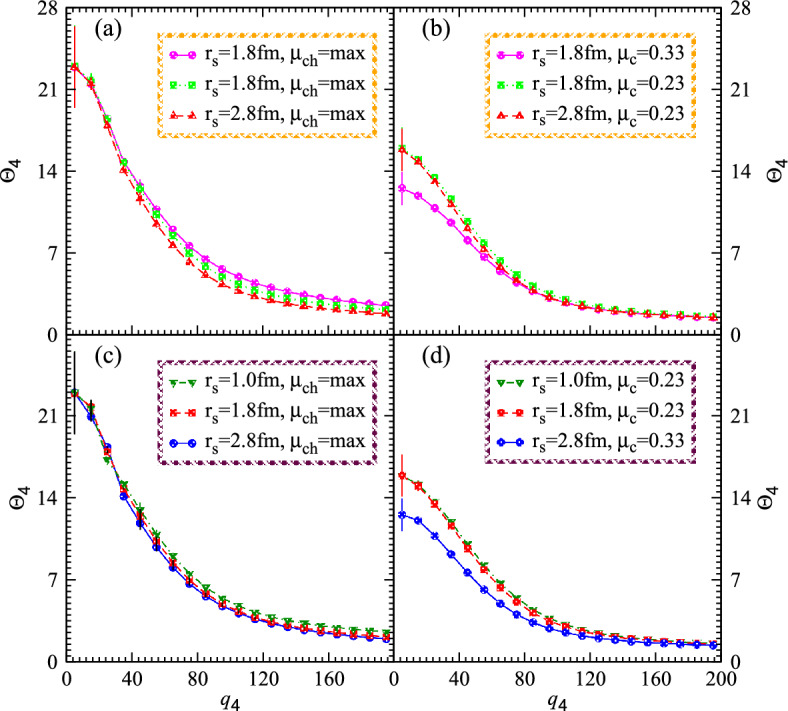
Nonlinear higher order correlations versus relative momenta for the nonlinear granular systems with different radii and chaos peculiarities.

Fig. 6 demonstrates the nonlinear higher order correlations $\Theta _4$ versus relative momenta $q_4$ for the nonlinear systems with different radii and chaotic fractions. The top panels a and b elucidate the correlations for the chaos and partially chaos-coherence emissions, respectively. The results show that the correlation touches the chaos limit at the intercept for the entire chaos nonlinear systems for all the radii and such correlations decrease gradually with increasing the relative momenta due to the isolation of interferences. It is more intriguing to mention that the left top and bottom panels a and c represent the source size at the large relative momenta with chaos emissions and the chaos characteristics at the small momenta measure the system size in the finite relative momenta. Hence the results of the large radii touch the coherent limit more frequently than that of the small systems and these results are more meaningful for determining the size as well as the chaos-coherence characteristics of the nonlinear systems in order to use them in pragmatic applications.

On the other hand, the partially chaos nonlinear systems expressed in the top and bottom panels b and d, respectively. The results exhibit an obvious suppression at the intercept in the presence of coherent emissions and increase the suppression with increasing the relative momenta. Hence correlations increase with decreasing the coherent fraction and increasing the chaos emissions as shown in the right panels of the aforementioned figure. Particularly, it can be seen that the correlations approach their chaotic limit under the presence of entirely chaotic particulate emergence of small droplets. Thus the right panels show that the correlation functions for cohesiveness droplets minimize extensively as the coherence increases and the results of the chaotic emissions within the nonlinear system enhanced with consistent to tiny droplets at the intercepted. It is also obvious that the radii of the little droplets described their propagations at high relative momenta and caused such meaningful results that describe the enormous tails on average momenta which indicate droplet radii whereas their small width measures larger droplet radii.

In particular, the present figures investigate the dynamic behavior of new types of nonlinear fractional dynamical systems with granular and finite chaos emissions. The new chaotic systems are modeled from well-known chaotic systems with density matrices and ordinary derivatives. We presented unique methodologies numerically to simulate the study of nonlinear systems as fractional systems that include chaos-coherence peculiarities. We concluded from the chaotic parameters figures and phase properties that the studied systems can display chaotic attractors and thus the research examines the possibility of chaos in fractional models with coherence exponents. The variations in the rich properties and complex dynamics of the proposed fractional models due to the presence of many possible options for the order function recommended the implementation of the studied generalized derivatives and the use of the presented method in fractional calculus.

**Figure 7:**
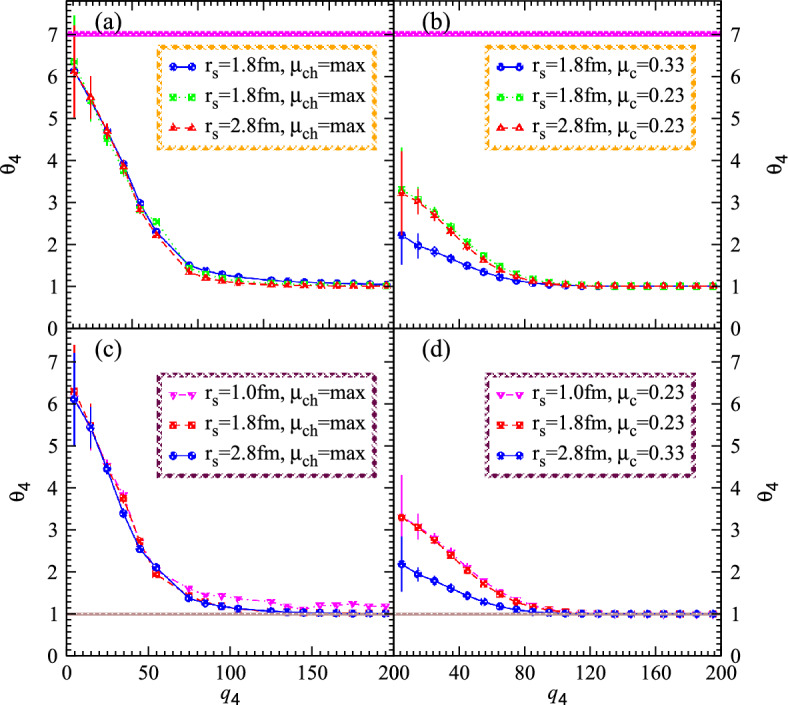
Nonlinear genuine higher order correlations versus relative momenta for the nonlinear systems with different radii and chaos-coherence characteristics within partially chaos emanations.

Furthermore, such kind of nonlinear correlation functions reveal the quadrat symmetrization and isolate the lower order interferences called the nonlinear genuine higher order symmetrization represented by $\theta _4$ as shown in Fig. 7. The top solid thick lines represent the chaotic limit of seven for ideal nonlinear chaotic systems. The interferences for the chaotic and partially chaotic coherent droplets are shown in the left and right panels, respectively. Therefore $\theta _4$ touches the chaos limit of seven in entirely chaotic circumstances of ideal chaotic systems as shown in the left top-bottom panels. The results are suppressed gradually during the coherence emissions from the tiny droplets since coherent droplets exhibit the peculiarities to emanate the coherence emissions. These intriguing correlations within the small droplets elevated at the point of intercept as the chaos emission rises and vice versa. The causes of such a scenario are apparent: as the percentage of coherence decreases the corresponding chaos of granular environment systems starts to increase. Significant condensing possesses a distinct relation with the coherence which leads to decrease in the normalized chaos parameters. The width of the slopes appears wider at smaller droplet diameters and less extensive at broad droplet radii with the substantial relative momenta allowing us to investigate the pretty nonlinear granular structure with greater precision than those of the lower order interferences.

Given these considerations, the objective of our specific research is to pave the path for distinctive multiple disciplines and ambitious investigations centered on sophisticated mathematical simulation and computational foundations. As a result, we anticipate putting forward theoretical computational, combining mathematical analyses, applied dimensions, procedures, experimental strategies, modeling, investigations, evaluations assessments, computerized assistance translations, technology for computing and so on to investigate the importance of creative methods and strategies in contemporary nonlinear systems alongside associated various realms.

The key parameters investigate the system structure with dividing the higher order correlators into pair interferences. Hence the normalizing of the fourth order interferences for nonlinear systems computes the chaotic-coherent percentage and thus certain computations overcome the influence of the Fourier phase which demonstrated as

28 $$\begin{aligned} w_{4g}{(q_4)}=\frac{\theta _4(q_4)-1}{(1-\upsilon _r)\sqrt{\Omega ^g(4,1)\Omega ^g(4,3)\Omega ^g(3,2)\Omega ^g(2,1)}}. \end{aligned}$$

Moreover, the normalized chaotic correlation coefficients of the chaotic variables can be influenced by system characteristics and appear very intriguing in the current research. Such computations can be demonstrated as follows: $\beta _{2}^{g}=(1-\upsilon _r)$, $\beta _{3}^{g}={(1-2\upsilon _r)}/{\sqrt{(1-\upsilon _r)}}$, $\beta _{4}^{g}={(1-2\upsilon _r)(1-3\upsilon _r)}/{(1-\upsilon _r)}$. In this instance, $\upsilon _r$ stands for the reciprocal of the droplet multiplicities. Therefore the normalization stochastic parameter interception essentially follows the nonlinear phenomena exhibits the pragmatic applications as $\beta _4^{p}={1+4\upsilon _c}/{(1+2\upsilon _c)^{2}}$. It must become clear that the coherent parameters vary with particular $\upsilon _c$ which specifies the proportion of coherent-chaotic systems in order to investigate various characteristics of hybridization systems within collective phenomena.

**Figure 8:**
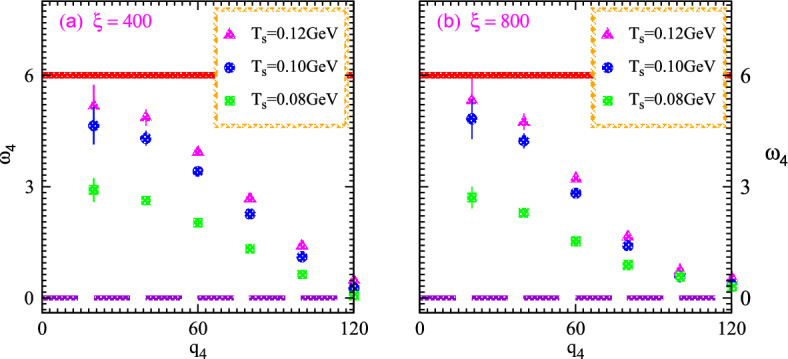
Normalized higher order chaotic correlators $\omega _4$ versus relative momenta $q_4$ for nonlinear systems at different values of the size parameters 0.35 (left) and 0.40 (right) possess various multiplicities four and eight hundred with the variation of temperatures $T_s$.

The objective of the current study is to investigate the distinctive characteristics of nonlinear system development caused by the influence of chaos-coherence characteristics. Equations involving differential can rigorously characterize the substantial ingredients as well as the multiplicity of the emanating systems which elucidate quantum scientifically normalized correlation factors. We analyze the momentum dependence normalized chaotic parameters along with the partial chaos sources and propose that the nonlinear mechanisms are composed of hydrodynamic clumps that exhibit partial chaotic characteristics at various temperatures and momentum domains. We present the results of correlations for second, third and fourth-order parameters versus analogous momenta at various temperatures. The relations between the coherent-chaotic emissions and their partial catastrophic source are thoroughly examined. The mixed-coherence interactions are ideally considered to demonstrate the coherence corrections that occurred through an effective investigation. With comparing observed chaos to coherence anticipated relationships, the coherence appeared to be negligible during higher momenta. Moreover, we also present our findings with chaotic-coherence implementations within the significant degrees of coherence at lower temperatures.

Fig. 8 illustrates the normalized chaotic parameter $\omega _4$ versus relative momentum $q_4$ for the nonlinear systems that expand and possess distinct multiplicity and temperatures $T_s$. Such nonlinear systems exhibit particle multiplicities, four and eight hundred within the specified source sizes or characteristics length of 0.40 and 0.35 as shown in the right and left panels, respectively. The small and large systems are represented by the left and right panels, respectively. The solid line at the top of two panels represents the chaotic limit in the case of four particle emissions and these lines indicate the chaotic limit six in the whole regime of relative momenta. The normalized correlations increase gradually with rising the temperature at constant relative momenta. Such intriguing results investigate the influence of condensation at various temperatures because the specific temperature possesses a relation with the particles kinetic energy. The particles kinetic energy increases with increasing temperature and such particles exhibit enough energy to overcome the energy gaps even in the case of small sources. One can also observe that the correlations show the suppression with increasing the relative momenta at all the temperatures. Such results explore the intrinsic peculiarities of the systems since the identical momenta particles have strong quantum interferences because identical momenta particles possess zero relative momenta and show strong correlation characteristics.

Furthermore, both panels demonstrate that the correlations of the large source elucidate strong correlations and moderate coherence in all the regimes of relative momenta at a constant temperature even though the large source possesses a double multiplicity than that of the small one. These intriguing results appear because the large system of particles exhibits small energy gaps due to large characteristics and thus particles acquire suitable energy to cross the energy gaps from ground to excited states.

**Figure 9:**
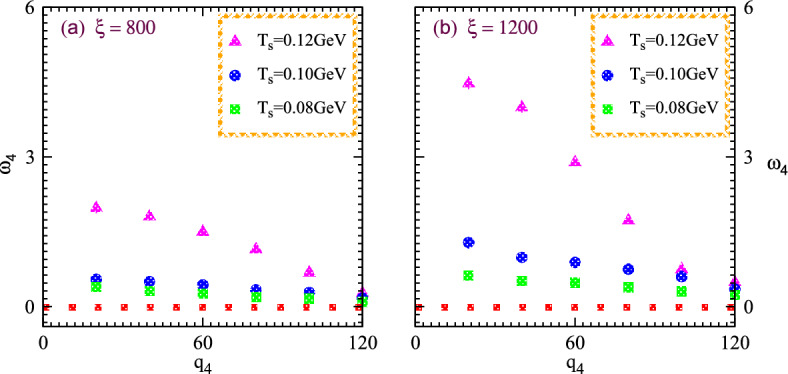
Nonlinear normalized higher order chaotic parameter $\omega _4$ versus relative momenta $q_4$ for nonlinear systems exhibit two different values of the source sizes parameters at particle multiplicities eight and twelve hundred with the variation of temperatures $T_s$.

Fig. 9 demonstrates the normalized parameters $\omega _4$ for partially chaotic emissions versus relative momenta $q_4$ within the nonlinear systems exhibiting two different values of the explored parameter at various particle multiplicities $\xi$ and the temperatures $T_s$. One can note that the results show the nonlinear system peculiarities with two horizontal panels for small and large systems, respectively. The significant suppression arises with increasing multiplicities at constant temperature and relative momenta due to the influence of coherence.

Normalized stochastic correlator increases with increasing the temperature at fixed volume and relative momenta as shown in the right panel. The declination patterns appear in the normalized correlation considerably at large relative momenta for both systems during the nonlinear phenomena within the dominance of coherence and absence of quantum interferences. Results show that the normalized chaotic parameter tends to decrease as temperature increases.

In particular, nonlinear systems with small scales possess a high condensation degree and wide energy gaps. Such condensations possess a certain relation to the particles overlapping at a particular temperature when associated wavelengths are in order of the inter-particle spacing which start to overlap and produce meaningful condensation. Therefore those systems which having lowering temperatures with constant volume and particle multiplicities exhibit condensation due to a deficiency of kinetic energy. Correspondingly the considered particles with lower kinetic energy perpetuate in the ground states and thus the lower momenta particles also produce peculiar condensation which influences the normalized correlation at small relative momenta. The characteristic length which measures the parameter 0.35 demonstrates significant condensation than that of the large system which is measured by the parameter 0.40 due to large size and narrow energy gaps of the nonlinear systems.

**Figure 10:**
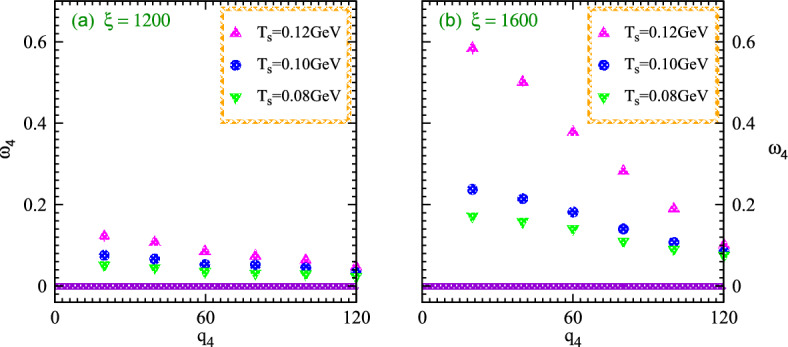
Normalized chaotic correlator $w_4$ versus relative momentum $q_4$ at higher order for the nonlinear systems of two sources with parameters contained multiplicities twelve and sixteen hundred with the variation of temperatures $T_s$.

Fig. 10 elucidates the normalized higher order nonlinear chaotic correlator $\omega _4$ versus relative momentum $q_4$ for the nonlinear systems with two source sizes possess the distinct multiplicities and temperatures to probe the coherence-chaos influences within the quantum interferences at high particle multiplicities. The considered system exhibits particle multiplicity of twelve hundred for the small size or characteristics length 0.35, and sixteen hundred for a large one with size parameter 0.40 as shown in the right and left panels, respectively.

It is meaningful to discuss that the $\omega _4$ chaotic parameter declines gradually with increasing the relative momenta at constant temperature and multiplicity in the specified regimes. The intriguing results show negligible interferences at large relative momenta because the particles have significant momentum at wide relative momenta and thus the quantum correlation characteristics disappear considerably. The normalized chaotic attractors increase with increasing the system temperature and vice versa at constant relative momenta and size of the nonlinear systems. Moreover, such nonlinear correlations suppress with increasing the multiplicities of particles. Since high multiplicity at a specific temperature contains wavelength that starts to condense and form the condensation which reduces the correlation influences and thus the corresponding normalized chaotic parameter shows their declination at high multiplicity and lower temperature.

The aforementioned figures demonstrate that the normalized chaotic correlator $\omega _4$ for the systems exhibits multiplicities of sixteen, twelve and eight hundred with large parameter of 0.40. Such meaningful correlator increases gradually with decreasing the multiplicities and increases with increasing the temperatures at specified relative momenta. These prodigious influences elucidate that the system with maximum particles exhibits a substantial coherence effect at curtailed momenta and temperature. Such parameters acquire the chaotic limit six at the intercept with increasing the temperature and decreasing the multiplicities significantly as shown in the aforementioned figures. The causes of such nonlinear phenomena are quite obvious that the particles at higher temperatures contained sufficient energy and thus the effect of quantum coherence becomes negligible which can vanish at higher temperatures. Therefore the normalized chaotic parameters accomplished their chaotic limit at zero relative momenta.

**Figure 11:**
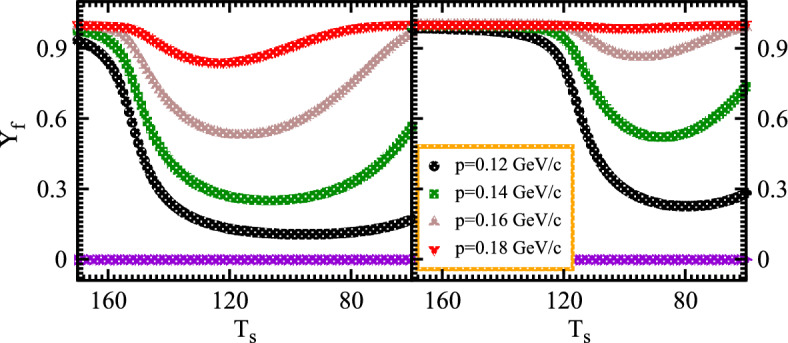
Normalized chaotic correlation $Y_f$ at higher order versus temperature for the nonlinear small and large systems contained twelve and sixteen hundred multiplicities at various momenta.

Fig. 11 elucidates the nonlinear system characteristics in order to investigate the consequences of coherent emission morphology on the measurement of normalized chaotic parameters at finite momenta. Such parameters with different system sizes possess various multiplicities and momenta at different temperatures. It seems more meaningful to discuss the chaotic parameter which continuously decreases with decreasing the temperature and hence the corresponding multiplicity increases within the fixed system volume. Despite small multiplicities at lower temperatures and momenta systems, an environment with extra particle multiplicities retains excess coherence that dominates the chaotic limit at various temperature regimes. The reasons for such results are very intriguing and meaningful for experimental applications. The parameter expands steadily as the momenta increase constantly over time and eventually reaches the state of maximum chaos at elevated temperatures as shown in both panels.

Specifically, it can observed that these chaotic variables at higher levels are suppressed dramatically with lower temperatures even possessing massive high momenta. Therefore such intriguing results indicate that the chaotic parameters exhibit meaningful normalized chaos variables at higher levels to investigate the characteristics of chaotic fluxes and wave propagations. Furthermore, the large nonlinear systems possess a smaller density than that of the small systems which differentiates the condensation for systems at various momenta. Such condensation suppresses the normalized chaotic correlation parameters significantly at the regimes of lower momenta.

In particular, the considered technique examined the chaos-coherence properties of the nonlinear systems and thus the higher order chaotic correlators depend on the system sizes to explore the intrinsic peculiarities. The lower order chaotic attractor obtained and such multiplication represented to demonstrate the collective phenomena in physical systems to develop the entanglements in the nonlinear systems. Thus the density factor appeared significant in probing the corresponding results and the chaotic-coherent components of the distribution functions associated with the evaluated multiplicities. The influence of the variable-order exponents on the dynamics of the considered chaotic systems also explored. This current section confirms that chaos does indeed exist in fractional systems with variable-order fractional derivatives.

## Summary and conclusion

This study investigated the dynamic behavior of new types of nonlinear fractional dynamical systems with chaotic parameters and chaos-coherence exponents. The new chaotic systems are modeled and presented an efficient techniques to numerically simulate the studied fractional system that includes chaos and coherence fractions. We concluded from the pictured chaotic parameters diagrams and phase that the studied systems can display chaotic attractors. The used derivative is influenced by the coherence exponents which hold the memory of the derivative order and, so offering an effective tool in building fractional models. The study confirms the possibility of chaos in fractional models with specified exponents. The variations in the rich properties and complex dynamics of the proposed fractional models, due to the presence of many possible options for the order function recommended the implementation of the studied generalized derivatives and the use of the presented methods in fractional calculus.

In particular, the investigation of the nonlinear systems propounded an intriguing challenge in the high dimensional analysis and the results primitively exhibit the correlations at higher temperatures-momenta regimes in order to explore the intrinsic peculiarities of nonlinear phenomena in physical and biological systems. The influences of condensation suppress the chaotic parameters within the particular systems and the exploration of such collective phenomena deals with interferometry through the quantum applications to probe the nonlinear system characteristics. The current study used meaningful methodologies and extended it to measure the isotropic sources characteristics which have acknowledged the presence of quantum chaos and coherence at lower temperatures. Particularly, the density matrix within numerical normalization expansion techniques demonstrates the coherence domination over quantum correlations at defined temperatures-momenta regimes. As a result, our research aims to explore the direct methods to elucidate the peculiarities of the complex systems with the existence of excited-ground states for the intermediate regimes of momenta.

In this research work, the nonlinear system under the coherence morphologies with coherent-chaotic characteristics are demonstrated and the pragmatic peculiarities described to discuss the internal characteristics of the systems with different parameters. We elucidated the density dependence coherence-chaotic emissions and explored the condensation within momentum-temperature regimes which probed the system geometry at specific momenta. The techniques accomplished subsist intelligible, dependable and categorical to correlate in various quantum phenomena. The quintessential propensities of the peculiarities of the nonlinear system are given in figures, tables and the consequences are enormously consistent pertinacious with the droplets and ascendance of the temperature regimes to harness in engineering-medical fields. The considered methodologies are particularly constructive within the presented aspects of the produced nonlinear systems and such a meaningful scenario investigates the variation in the temperature-energy regimes at the particular systems dimensions. Such scientific demonstration attained captivating potential to invigorate scientists, engineers and scholars from multifarious fields.

## Data Availability

The authors confirm that the data of this research are available within the manuscript and requests for materials should be addressed to the corresponding author.
